# Identification of SNPs associated with methotrexate treatment outcomes in patients with early rheumatoid arthritis

**DOI:** 10.3389/fphar.2022.1075603

**Published:** 2022-11-17

**Authors:** Shrikant S. Kolan, Gaoyang Li, Franco Grimolizzi, Joe Sexton, Guro Goll, Tore K. Kvien, Nina Paulshus Sundlisæter, Manuela Zucknick, Siri Lillegraven, Espen A. Haavardsholm, Bjørn Steen Skålhegg

**Affiliations:** ^1^ Department of Nutrition, Division of Molecular Nutrition, Institute of Basic Medical Sciences, University of Oslo, Oslo, Norway; ^2^ Center for Treatment of Rheumatic and Musculoskeletal Diseases (REMEDY), Diakonhjemmet Hospital, Oslo, Norway; ^3^ Institute of Clinical Medicine, University of Oslo, Oslo, Norway; ^4^ Department of Biostatistics, Oslo Centre for Biostatistics and Epidemiology, Institute of Basic Medical Sciences, University of Oslo, Oslo, Norway; ^5^ Department of Health Management and Health Economics, Institute of Health and Society, University of Oslo, Oslo, Norway

**Keywords:** methotexate, rheumatoid arthritis, single nucelotide polymorphisms, clincal outcomes, association

## Abstract

Methotrexate is one of the cornerstones of rheumatoid arthritis (RA) therapy. Genetic factors or single nucleotide polymorphisms (SNPs) are responsible for 15%–30% of the variation in drug response. Identification of clinically effective SNP biomarkers for predicting methotrexate (MTX) sensitivity has been a challenge. The aim of this study was to explore the association between the disease related outcome of MTX treatment and 23 SNPs in 8 genes of the MTX pathway, as well as one pro-inflammatory related gene in RA patients naïve to MTX. Categorical outcomes such as Disease Activity Score (DAS)-based European Alliance of Associations for Rheumatology (EULAR) non-response at 4 months, The American College of Rheumatology and EULAR (ACR/EULAR) non-remission at 6 months, and failure to sustain MTX monotherapy from 12 to 24 months were assessed, together with continuous outcomes of disease activity, joint pain and fatigue. We found that the SNPs rs1801394 in the *MTRR* gene, rs408626 in *DHFR* gene, and rs2259571 in *AIF-1* gene were significantly associated with disease activity relevant continuous outcomes. Additionally, SNP rs1801133 in the *MTHFR* gene was identified to be associated with improved fatigue. Moreover, associations with *p* values at uncorrected significance level were found in SNPs and different categorical outcomes: 1) rs1476413 in the *MTHFR* gene and rs3784864 in *ABCC1* gene are associated with ACR/EULAR non-remission; 2) rs1801133 in the *MTHFR* gene is associated with EULAR response; 3) rs246240 in the *ABCC1* gene, rs2259571 in the *AIF-1* gene, rs2274808 in the *SLC19A1* gene and rs1476413 in the *MTHFR* gene are associated with failure to MTX monotherapy after 12–24 months. The results suggest that SNPs in genes associated with MTX activity may be used to predict MTX relevant-clinical outcomes in patients with RA.

## 1 Introduction

Rheumatoid arthritis (RA) is a chronic autoimmune disease characterized by systemic inflammation manifested in multiple joints (Smolen and Aletaha, 2006). In the absence of early and effective treatment, the condition can lead to permanent damage, disability, reduced quality of life and life expectancy (Aletaha and Smolen, 2018). The management of RA has undergone significant changes leading up to current treat-to-target strategy, which aims for remission or at least low disease activity (Aletaha and Smolen, 2018; Smolen et al., 2020). Current international guidelines endorse methotrexate (MTX) as a first-line therapy because of its efficacy, cost, and availability of long-term safety data (Gispen et al., 1987; Klareskog et al., 2004; Salliot and van der Heijde, 2009). Despite the effectiveness and clinical utility of MTX, approximately 50%–60% of the patients do not show significant benefit from the treatment due to lack of efficacy or adverse effects (Goekoop-Ruiterman et al., 2007; Hørslev-Petersen et al., 2014; Nam et al., 2014; Emery et al., 2015).

Factors shown to be associated with MTX non-response may be patient-related such as gender and history of smoking and disease-related as duration and severity (Saevarsdottir et al., 2011; Gosselt et al., 2021). Furthermore, levels of anti-citrullinated protein (ACPA) and rheumatoid factor (RF) are considered relevant for MTX responses (Martínez et al., 2020; Maciejewski et al., 2021; Wu et al., 2021). Finally, 15%–30% of variation in drug response are thought to be attributable to patient’s genetic factor or single nucleotide polymorphisms (SNPs) (Eichelbaum et al., 2006).

**FIGURE 1:**
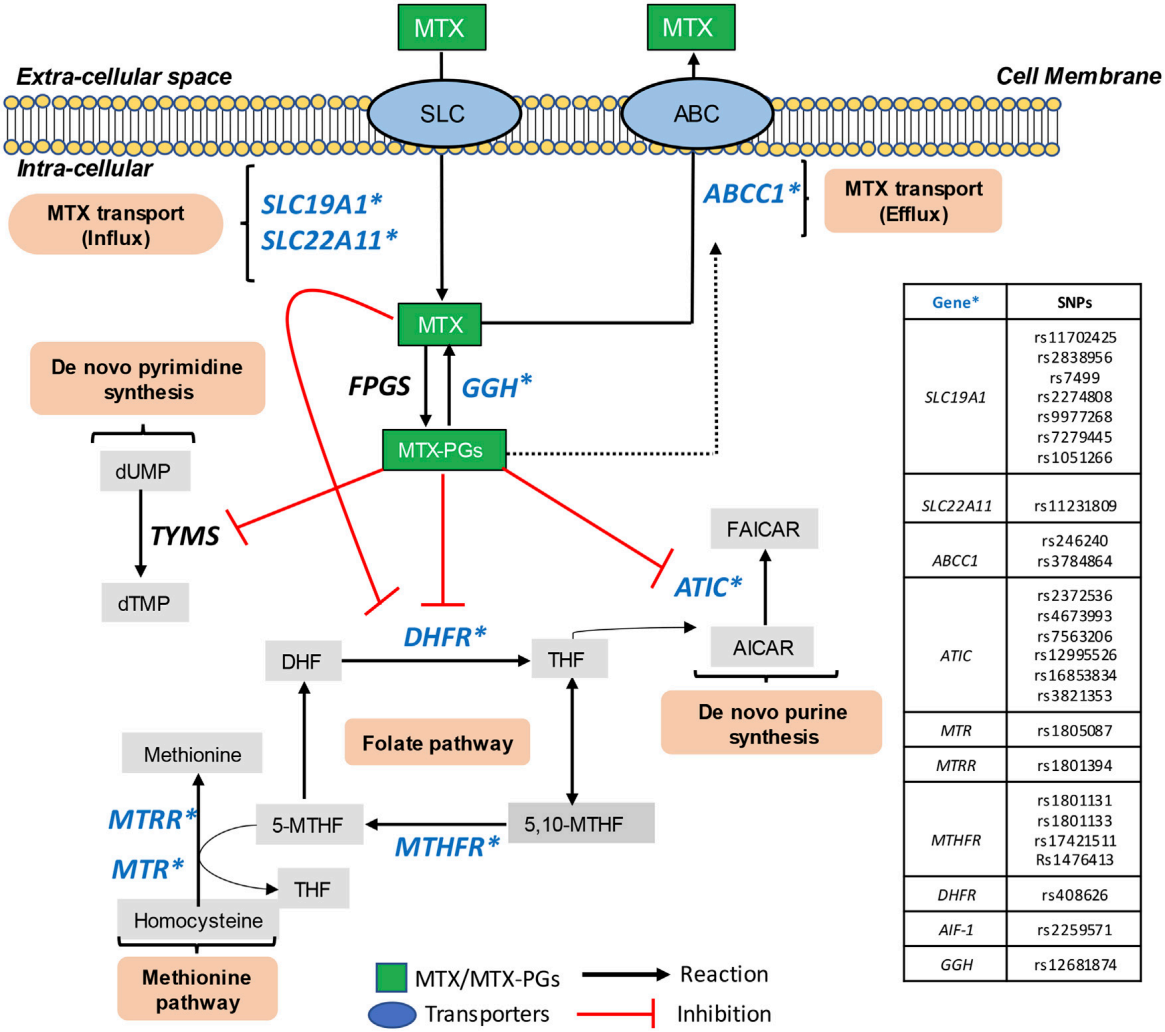
Metabolic pathways of intracellular metabolism of MTX. MTX, Methotrexate; SLC, solute carrier; ABC, ATP-binding cassette transporters; FPGS, folylpoly-γ-glutaminase synthetase; GGH, γ-glutamyl hydrolase; MTX-PGs, methotrexate polyglutamate; DHFR, dihydrofolate reductase; DHF, dihydrofolate; THF, tetrahydrofolate; 5,10-MTHF, methyltetrahydrofolate cyclohydrolase; 5-MTHF, L-methylfolate; MTR, methionine synthase; MTRR, methionine synthase reductase; MTHFR, methylenetetrahydrofolate reductase; TYMS, thymidylate synthase; dUMP, deoxyuridine monophosphate; dTMP, deoxythymidine monophosphate; ATIC, 5-aminoimidazole-4-carboxamide ribonucleotide formyltransferase; AICAR, 5-aminoimidazole-4-carboxamide ribonucleotide; AMP, adenosine monophosphate; FAICAR, 5-formylaminoimidazole-4-carboxamide ribonucleotide; IMP, inosine monophosphate; AMP, adenosine monophosphate; AIF-1, allograft inflammatory factor 1 and GGH, gamma-glutamyl hydrolase. SNPs included in the current study are marked with blue font and star (*).

MTX efficacy has been linked to polymorphism in genes encoding proteins regulating MTX influx, efflux, metabolism, and effector pathways (Figure 1) (Qiu et al., 2017). To this end, cellular uptake of MTX is mediated by reduced folate carrier protein 1 (RFC1, also called SLC19A1) while the ATP-binding cassettes (ABCC) family transporters mediate its cellular efflux (Moscow et al., 1995; Ranganathan and McLeod, 2006; Hider et al., 2007). Once internalized, MTX is reversibly converted to active MTX polyglutamates (MTX-PGs), controlled by the polyglutamation-deconjugation cycle mediated by folypolyglutamate synthetase (FPGS) and gamma-glutamyl hydrolase (GGH) respectively (Chabner et al., 1985). Polyglutamated MTX (MTX-PG) competitively inhibits dihydrofolate reductase (DHFR) and other enzymes such as methylenetetrahydrofolate reductase (MTHFR) contributing to the anti-folate effects of MTX, although not by direct inhibition (Kooloos et al., 2010; Malik and Ranganathan, 2013). Finally, MTX-PG inhibits thymidylate synthase (TYMS) and AICAR (5-aminoimidazole-4-carboxamide ribonucleotide) formyltransferase (ATIC), which contribute to the intracellular accumulation of adenosine (Kooloos et al., 2010; Inoue and Yuasa, 2014; Zhu et al., 2014).

The association between polymorphisms in these genes and MTX response have been described (Owen et al., 2013a; Lv et al., 2018; D’Cruz et al., 2020). However, due to heterogeneous study populations and replication failure, different conclusions have been reported. Thus, the identification and development of clinically effective SNP biomarkers for predicting MTX sensitivity remains a challenge. It is therefore a need to replicate and validate candidate SNPs in various and large patient cohorts. In the present study, we aimed to investigate the associations between 23 SNPs and broad clinically relevant outcomes to assess their contributions to MTX efficacy in a cohort of 224 Norwegian patients with RA who were naïve to MTX and included in the ARCTIC trial (Haavardsholm et al., 2016). The outcomes assessed were categorical outcomes, including ACR/EULAR Boolean non-remission, DAS-based EULAR non-response and failure to MTX monotherapy as well as continuous outcomes including disease activity, fatigue and joint pain. We found that variations in genes encoding key components regulating MTX influx, efflux, metabolism pathways may be used to evaluate clinical outcomes in patients with RA.

## 2 Materials and methods

### 2.1 Subjects

The current ARCTIC_SNPs study included 224 patients, a sub-cohort from the original ARCTIC clinical trial (Haavardsholm et al., 2016). The ARCTIC trial was a 24-month randomized, open label, prospective, national, multi-centric study (11 centers in Norway) conducted in accordance with the Declaration of Helsinki and the International Conference on Harmonization Guidelines for Good Clinical Practice. Informed consent was taken from all the study subjects. All patients must have satisfied the following criteria: 1. Age between 18 and 75 years; 2. Fulfillment of the 2010 American College of Rheumatology (ACR)/European League against Rheumatism (EULAR) criteria for RA; 3. No prior treatment with DMARD including MTX; 4. Disease duration less than 2 years and 5. Indication for DMARD treatment. Patients with major comorbidities and abnormal liver or kidney function were excluded from the study (Haavardsholm et al., 2016). After diagnosis, all patients received MTX together with tapering dose of prednisolone.

### 2.2 Outcome assessment

Sensitivity to MTX treatment was evaluated based on both categorical and continuous outcomes. The primary categorical outcome for the study was that the patient did not achieve ACR/EULAR Boolean remission at 6 months of follow-up (‘non-remission’). The patient must satisfy the following criteria in order to achieve ACR/EULAR boolean remission: Ritchie Articular Index ≤1 (RAI≤1); Swollen Joint Count ≤1 (SJC44 ≤ 1); C-Reactive Protein ≤1 (CRP≤1); and the Patient’s Global Assessment ≤1 (PGA≤1) (Felson et al., 2011). The secondary categorical outcome was according to disease activity scores (DAS) based EULAR non-response at 4 months of follow-up. This classifies patients into good, moderate- and non-responders based both on level and changes in DAS (Supplementary Table S1) (Fransen and van Riel, 2005). Good responders were denoted as favorable outcome and moderate/non-responders were considered as unfavorable outcome. The other secondary categorical outcome was the failure to sustained MTX monotherapy defined based on persistence of MTX monotherapy. Patients with MTX monotherapy at both 12 months and 24 months reflect favorable outcome otherwise unfavorable. In addition, continuous outcomes include DAS, clinical disease activity index (CDAI) (Aletaha et al., 2005), simplified disease activity index (SDAI) (Smolen et al., 2003), joint pain and fatigue at 4 or 6 months, as well as improvement of these measures from baseline.

### 2.3 Selection of single-nucleotide polymorphisms

Twenty-five SNPs in nine candidate genes (marked in blue in Figure 1) were selected based on literature attending to their putative contribution in MTX metabolic pathways and mechanism of transport and action. They included, genes involved in MTX cellular influx (*SLC19A1 and SLC22A11*; -efflux (*ABCC1*), –and polyglutamate formation (*GGH*), in addition to the folate- (*DHFR and MTHFR*) and methionine pathways (*MTR and MTRR*), as well as *de novo* purine synthesis (*ATIC*) and finally, inflammatory cytokines (*AIF-1*) (Qiu et al., 2017) (Supplementary Table S2).

### 2.4 DNA isolation and genotyping

Genomic DNA was extracted from full blood by using GeneJET Whole Blood Genomic DNA Purification Mini Kit as per manufacturer’s instruction (ThermoFisher, Waltham, United States). Total genomic DNA was quantified and then analyzed for purity using the NanoDrop 1000 Spectrophotometer v3.7 (Thermo Fisher Scientific, Waltham, United States). Sequenom^®^ Assay Designer 3.1 software was used for primers design, and samples were genotyped using the Sequenom iPLEX^®^ MassARRAY platform according to the manufacturer’s instructions (OE Biotechnology Co., Ltd., Shanghai, China).

### 2.5 Statistical analysis

As quality control, SNPs were excluded from analysis when genotyping success rates were less than 95% or when minor allele frequency (MAF) was less than 5%. Moreover, deviation of SNPs from Hardy-Weinberg equilibrium (HWE) was assessed by a Pearson’s chi-squared test with a significance threshold of *p* < 0.05 and those SNPs were excluded. Demographic and clinical categorical variables were summarized by frequency and percentage while continuous variables were summarized by median and interquartile range (IQR, 25–75th percentile). Differences in the categorical variables between subgroups were analyzed by Pearson’s chi-squared test and Mann–Whitney U test for continuous variables. To analyze the associations between SNPs and categorical clinical outcomes, both univariate and multivariate analyses were performed using three genetic models. These included the genotype model denoted as major homozygote vs. heterozygote vs. minor homozygote, the recessive model denoted as major homozygote + heterozygote vs. minor homozygote and the dominant model which was major homozygote vs. heterozygote + minor homozygote). To implement univariate analysis, the odds ratio (OR) (95% confidence interval) of each SNP was calculated from cross-tabulation and Pearson’s chi-squared test, or Fisher exact test when expected case number ≤5, was performed. Haldane-Anscombe correction was adopted if the count number in a subgroup was zero when calculating OR. To conduct multivariate analysis, a logistic regression model was fitted with covariates age, gender, BMI and smoking in addition to the SNP variable, and the corresponding adjusted OR for each genotype was calculated from the estimated regression coefficient of the SNP variable in the model. To identify the association between SNPs and continuous clinical outcomes, Spearman’s rank correlation was used to calculate correlation coefficient ρ and *p*-values for each SNP encoded by counts of minor allele (major homozygote: 0 as reference, heterozygote: 1, minor homozygote: 2). Univariate and multivariate analysis by ordinary least squares linear regression (OLS) was employed to further explore the association between SNP and continuous endpoints, with age, gender, BMI and smoking as covariates in multivariate models. Results were considered statistically significant when two-sided *p*-values were less than 0.05. The adjustment of significant results for multiple testing by Bonferroni correction (significant level of 0.0022 corrected based on a group of hypotheses tested on 23 SNPs) was reported in all analyses on association between SNPs and outcomes. OLS is the only analysis where significance remains after Bonferroni correction. For other analyses we also chose to report the raw *p*-values (without multiple testing adjustment) because of the exploratory nature of the analyses and to better highlight nuances in the results that would be less visible after correction.

## 3 Results

### 3.1 Patient inclusion

**FIGURE 2:**
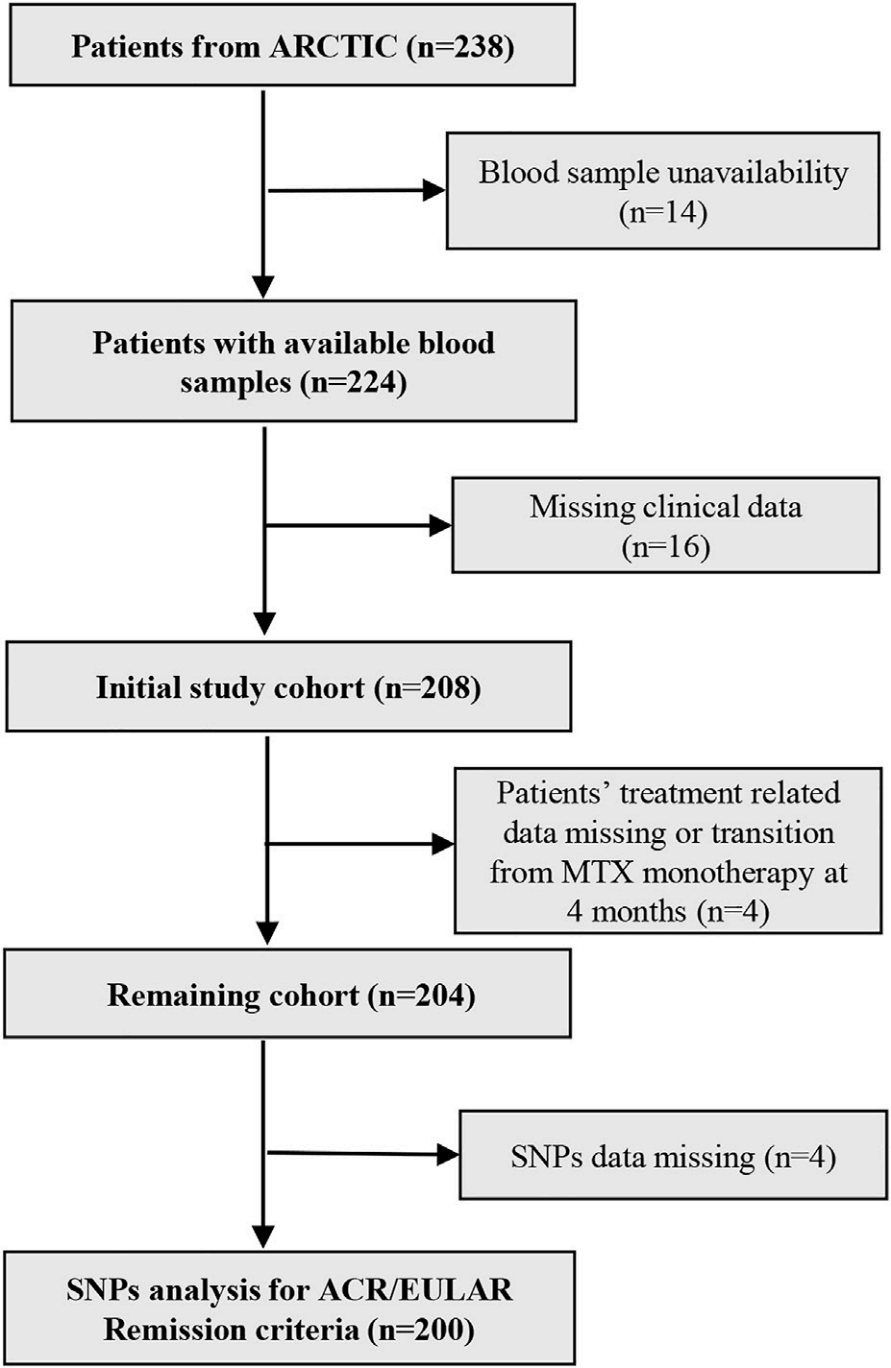
Flow chart for patient’s inclusion and exclusion criteria. N, number of patients; MTX, methotrexate; SNPs, Single nucleotide polymorphisms.

Out of 238 patients from the ARCTIC trial (Haavardsholm et al., 2016), 14 patients were excluded as blood-samples were missing, leaving 224 patients for the present study. Next, 16 plus 4 plus 4 patients were excluded due to missing clinical data, inadequate information on treatments or transition from MTX monotherapy at 4 months, and genotyping failure respectively. This resulted in inclusion of 200 patients in the present analysis (Figure 2).

### 3.2 Baseline characteristics

The demographic and clinical characteristics of the recruited are summarized in Table 1 and show that 78 (39%) were males and 122 (61%) were females with median age of 54 years. Seventy (35%) of the patients were never smokers, 86 (43%) were former smokers while 44 (22%) were active smokers at the time of RA diagnosis. The median body mass index (BMI) was 25.1. Moreover, the median of disease duration indicating months since first symptoms was 5.5 months while 144 (72%) and 168 (84%) of the patients were RF and anti-cyclic citrullinated protein (anti-CCP) positive, respectively. The median baseline value of CDAI and SDAI before treatment were 21 and 22, respectively. The median PGA and physician global assessment score (PhGA) at the baseline were 48 and 36 respectively, whereas erythrocyte sedimentation rate (ESR) and CRP for patients were 20 mm/h and 7 mg/L. Moreover, median value of joint pain, fatigue, patients reported outcomes measurement information physical function T-score (PROMIS-PF) and RA impact disease score (RAID) before treatment were 45.5, 37.5, 39.6, and 4.4 respectively. The median baseline value of DAS before treatment initiation was 3.32. Median start dose for treatment was 15 mg MTX/week at baseline.

**TABLE 1:** Characteristics of the patients enrolled in the study.

Characteristics	All patients (*n* = 200)	# Remission (*n* = 71)	# Non-remission (*n* = 129)	p-Value
	Median (25th-75th) and † number	Median (25th-75th) and † number	Median (25th-75th) and † number	
Patients related				
Age (years)	53.95 [42.4–62.7]	51.4 [39.1–63.0]	55.0 [44.4–62.7]	0.15
Gender (†)				0.26
Males (n)	78 (39%)	24 (34%)	54 (42%)	
Females (n)	122 (61%)	47 (66%)	75 (58%)	
BMI (kg/m2)	25.11 [22.75–28.13]	24.11 [21.87–27.49]	25.7 [23.44–28.41]	0.04*
Smoking (†)				0.7
Never Smokers (n)	70 (35%)	24 (34%)	46 (36%)	
Former Smokers (n)	86 (43%)	29 (41%)	57 (44%)	
Smokers (n)	44 (22%)	18 (25%)	26 (20%)	
Disease related				
Disease duration (months)	5.45 [2.8–9.9]	5.4 [3.2–11.9]	5.5 [2.7–9.5]	0.42
Anti-CCP (†)				0.51
Positive (n)	168 (84%)	58 (82%)	110 (85%)	
Negative (n)	32 (16%)	13 (18%)	19 (15%)	
RF-positive (†)				0.18
Yes (n)	144 (72%)	47 (66%)	97 (75%)	
No (n)	56 (28%)	24 (34%)	32 (25%)	
DAS	3.32 [2.56–4.1]	2.82 [2.27–3.45]	3.62 [2.81–4.45]	<0.001***
Ritchie Articular Index	7.0 [3.0–12.0]	4.0 [2.5–8.0]	8.0 [4.0–14.0]	<0.001***
SJC44	9.0 [4.0–14.0]	8.0 [3.5–13.0]	10.0 [5.0–15.0]	0.08
PGA	48.0 [28.75–69.0]	33.0 [20.5–52.0]	54.0 [38.0–75.0]	<0.001***
PhGA	36.0 [23.0–54.2]	30.0 [19.0–42.0]	41.0 [26.0–61.0]	<0.001***
ESR (mm/h)	20.0 [11.75–31.2]	16.0 [10.5–27.5]	22.0 [13.0–34.0]	0.02*
CRP (mg/L)	7.0 [3.0–18.0]	5.0 [2.0–8.5]	9.0 [4.0–19.0]	0.001***
Joint pain	45.5 [29.0–69.2]	32.0 [19.0–49.5]	53.0 [33.0–72.0]	<0.001***
Fatigue	37.5 [12.7–62.2]	26.0 [6.5–49.0]	47.0 [22.0–68.0]	<0.001***
PROMIS-PF	39.6 [33.2–44.7]	43.6 [37.5–48.6]	36.1 [30.5–41.6]	<0.001***
CDAI	21.0 [13.6–30.7]	16.9 [9.6–24.0]	24.2 [15.9–33.4]	<0.01**
SDAI	22.0 [15.1–32.1]	17.4 [10.0–24.2]	25.7 [17.5–36.3]	<0.001***
RAID	4.36 [2.8–5.8]	2.91 [1.95–4.2]	5.26 [3.76–6.6]	<0.001***
Treatment related (starting dose)				
Methotrexate (mg/week)	15.0 [15.0–15.0]	15.0 [15.0–15.0]	15.0 [15.0–15.0]	0.87

*p-value ≤ 0.05, **p-value ≤ 0.01, ***p-value ≤ 0.001.

Baseline clinical characteristics of recruited cohort and between remission group and non-remission group for SNPs analysis. # Clinical outcomes at 6-month to MTX treatment are defined according to ACR_EULAR remission criteria. Categorical variables (†) are expressed n = number and significance (*p*-value) is calculated by Chi-Square test. Continuous variables expressed as a median [25–75th percentile] and significance (*p*-value) is calculated by Mann-Whitney U test. Abbreviations: *n* = number of patients; RF, rheumatoid factor; Anti-CCP, Anti-cyclic citrullinated protein; Disease duration (months), Disease duration in months; BMI, body mass index; DAS, disease activity scores; SJC44, Swollen joint count based on 44 joints; PGA, patients global assessment score; PhGA, physician global assessment score; ESR, erythrocyte sedimentation rate; CRP, C reactive protein; Fatigue, Fatigue measured by visual analogue scale (0–100 mm); Pain, Pain measured by visual analogue scale (0–100 mm); PROMIS-PF, Patients reported outcomes measurement information physical function T-score, calculated by the sum of the values of the response to each question (range from 20 to 100); CDAI, clinical disease activity index; SDAI, simplified disease activity index; RAID, RA impact disease score.

### 3.3 Outcomes

At 6 months, 71 (35.5%), and 129 (64.5%) and patients were classified as remission and non-remission to MTX treatment, respectively, according to ACR/EULAR Boolean remission criteria. There were no statistically significant differences in categorical variables (gender/smoking/RF-status/anti-CCP status) and disease duration variable between the remission and non-remission groups. By contrast, the non-remission group had significantly higher BMI, disease activity composite scores, tender and painful joints assessed by RAI, PGA and PhGA values at the baseline. Patients not achieving remission at 6 months also showed significantly elevated levels of ESR, CRP, joint pain, fatigue and RAID at the baseline, as compared to remission group (see Table 1 for details). Finally, the median baseline value of PROMIS-PF for the non-remission group was significantly lower (indicating worse physical condition) as compared to remission group. For another two outcomes, the comparison of variables in two subgroups is shown in Supplementary Figure S1.

### 3.4 Single nucleotide polymorphisms selection

As depicted in Table 2 and Figure 1, a total of 25 SNPs were selected, based on their involvement in MTX uptake and metabolism pathways. In Table 2, the gene names and chromosome location of each SNP together with genotypic distribution of minor allele frequency (MAF) and Hardy-Weinberg equilibrium (HWE) are listed. Two SNPs, rs12681874 located in *GGH* and rs3821353 in *ATIC* were excluded due to failure in genotyping success rate (<95%) and non-agreement with HWE (*p* < 0.05), respectively, leaving 23 SNPs for the final analysis.

**TABLE 2:** Characteristics of the polymorphisms analyzed.

Gene name ( location)*	Pathway involved #	ID (SNPs)†	Major allele (M)	Minor allele (m)	MAF	MM/Mm/mm ∆	HWE-p value
*ATIC* [Chr 2]	Purine synthesis	rs2372536	C	G	0.30	108/89/23	0.52
		rs4673993	T	C	0.30	107/90/23	0.53
		rs7563206	C	T	0.45	66/107/47	0.79
		rs12995526	C	T	0.45	65/108/47	0.89
		rs16853834	C	T	0.16	156/56/8	0.32
		rs3821353**	G	T	0.27	124/73/23	0.02
*SLC19A1* [Chr 21]	MTX transporters	rs11702425	T	C	0.29	112/88/20	0.62
		rs2838956	A	G	0.41	76/104/40	0.67
		rs7499	G	A	0.38	86/100/34	0.57
		rs2274808	C	T	0.18	148/62/10	0.27
		rs9977268	C	T	0.17	155/57/8	0.33
		rs7279445	C	T	0.48	58/109/53	0.89
		rs1051266	G	A	0.41	74/109/37	0.88
*SLC22A11* [Chr 11]	MTX transporters	rs11231809	T	A	0.42	69/116/35	0.27
*ABCC1* [Chr 16]	MTX transporters	rs246240	A	G	0.16	151/64/5	0.80
		rs3784864	G	A	0.47	62/109/49	1.00
*DHFR* [Chr 5]	Folate	rs408626	A	G	0.46	62/111/47	0.89
*MTHFR* [Chr 1]	Folate	rs1801131	A	C	0.32	96/93/14	0.12
		rs1801133	C	T	0.31	108/87/25	0.27
		rs17421511	G	A	0.17	151/63/6	1.00
		rs1476413	C	T	0.28	107/100/13	0.13
*MTR* [Chr 1]	Methionine	rs1805087	A	G	0.20	136/79/5	0.14
*MTRR* [Chr 5]	Methionine	rs1801394	G	A	0.43	68/111/41	0.78
*AIF-1* [Chr 6]	Inflammatory cytokine	rs2259571	T	G	0.39	79/108/33	0.77
*GGH* [Chr 8]	MTX polyglutamation	rs12681874**	-	-	-	-	-

*Name of MTX metabolic enzymes and chromosome location; # Pathway regulated by MTX metabolic enzyme; † ID (SNPs) studied in the current analysis; Δ MM, homozygote for the common (major) allele (two major alleles); Mm, heterozygote (one major allele and one minor allele); mm, homozygote for the rare (minor) allele (two minor alleles). **SNPs were excluded due to violating HWE for rs3821353 and missing data for rs12681874. Chr, Chromosome; MAF, minor allele frequency calculated in studied population; HWE, Hardy-Weinberg equilibrium.

### 3.5 Single nucleotide polymorphisms associated with categorical outcomes post methotrexate treatment

**FIGURE 3:**
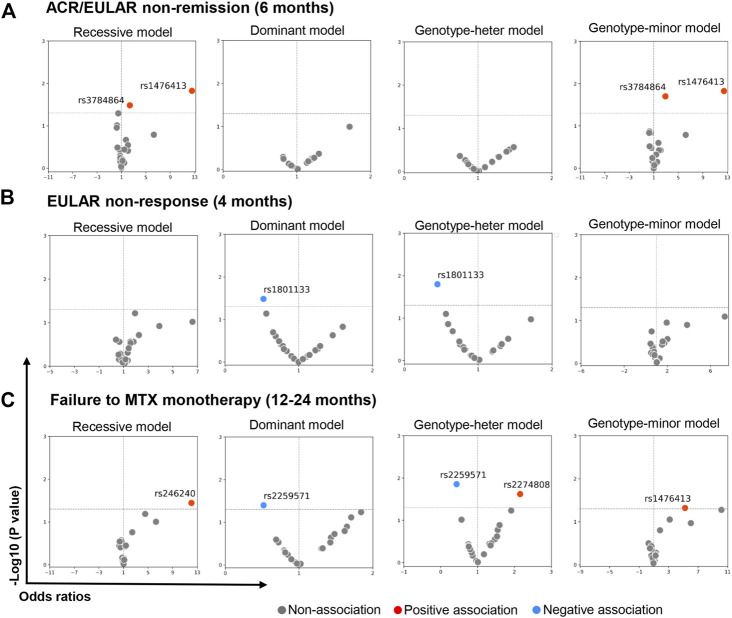
SNPs associated with MTX treatment inefficiency measured by categorical outcomes. Volcano figures showing SNPs significantly associated with MTX treatment inefficiency. Multivariate logistic regression (adjusted by gender, age, smoking, BMI) was implemented to calculate odds ratio and adjusted *p*-value (without Bonferroni correction) of each SNP. For those where case number in subgroups is zero, chi-square test was used and odds ratio was estimated by contingency table. MTX treatment inefficiency was measured by three criteria and denoted as ACR/EULAR non-remission at 6 months **(A)**, EULAR non-response at 4 months **(B)** and failure to MTX monotherapy from 12 to 24 months **(C)**. Recessive, dominant, genotype-heter and genotype-minor models were applied respectively. They are denoted as mm vs. MM + Mm (reference), mm + Mm vs. MM (reference), Mm vs. MM (reference) and mm vs. MM (reference) accordingly. The Bonferroni corrected *p*-value was estimated based on hypotheses tested on 23 SNPs to adjust significant results for multiple testing. The raw *p*-values (without Bonferroni correction adjustment) were also reported because of the exploratory nature of the analyses and to better highlight nuances in the results that would be less visible after correction. MM, major homozygote; Mm, heterozygote; mm, minor homozygote.

Three models, a genotype-, recessive- and dominant genetic model, were used in the present study to explore the association between SNP and classification outcomes. Pearson’s Chi-square test was used for univariate analysis of each SNP, and multivariable logistic regression model was applied with adjustment for gender, BMI, age, and smoking to assess potential confounding effects. We found that minor homozygote of two SNPs, TT in rs1476413 (C>T) in the *MTHFR* and AA in rs7884864 (G>A) in the *ABCC1* genes were associated with ACR/EULAR non-remission at 6 months in both the recessive and the genotype minor model (Figure 3A red dots, and Table 3A). No SNPs were significantly associated with EULAR non-response at 4 months (Figure 3B; Table 3B). By contrast, the minor allele T and heterozygote CT of SNP rs1801133 in gene *MTHFR* was significantly associated with EULAR response at 4 months in the dominant and genotype-heterozygote models, respectively (Figure 3B blue dot and Table 3B). Furthermore, the minor homozygote GG of rs246240, the heterozygote CT of rs2274808 and the minor homozygote TT of rs1476413 were all significantly associated with failure of sustained MTX monotherapy in the recessive, genotype-heterozygote and genotype-minor models, respectively (Figure 3C red dots and Table 3C). In addition to this, the minor allele G and heterozygote TG of rs2259571 was significantly associated with sustained MTX monotherapy in dominant and genotype-heterozygote model respectively (Figure 3C blue dots and Table 3C). SNPs found not associated with clinical outcomes are shown in Supplementary Table S3–5. However, it is worth mentioning that all these associations became insignificant when significance level was adjusted by Bonferroni correction.

**TABLE 3:** SNPs associated with MTX treatment inefficiency measured by categorical outcomes.

A. ACR/EULAR non-remission (6 Months)							
SNPS	Genotypes	non-Remission #N = 129	Remission #N = 171	*p*-value (Bonferroni corrected *p*-value)	Or (95% CI)	Adjusted *p*-value (Bonferroni corrected *p*-value)	Adjusted or (95% CI)
rs1476413 (C>T)	Recessive						
	CT + CC	119	71	0.015*(0.345)	Ref	-	-
	TT	10	0		12.56 (0.73–217.68)		
rs3784864 (G>A)	Recessive						
	GA + GG	95	61	0.045 (1)	Ref	0.033 (0.759)	Ref
	AA	34	10		2.18 (1.01–4.74)		2.37 (1.07–5.26)
rs147641**3** (C>T)	Genotype						
	CC	61	36	0.941 (1)	Ref	0.961 (1)	Ref
	CT	58	35	0.015 (0.345)	0.98 (0.55–1.76)	-	1.02 (0.55–1.86)
	TT	10	0		12.46 (0.71–219.04)		-
rs3784864 (G>A)	Genotype						
	GG	32	25	0.356 (1)	Ref	0.347 (1)	Ref
	GA	63	36	0.027 (0.621)	1.37 (0.70–2.66)	0.020 (0.46)	1.39 (0.70–2.75)
	AA	34	10		2.66 (1.10–6.40)		2.91 (1.18–7.15)

B. EULAR non-response (4 Months)							
SNPS	Genotypes	non-Responders #N = 76	Responders #N = 124	*p*-Value (Bonferroni CORRECTED *p*-value)	Or (95% CI)	Adjusted *p*-value (Bonferroni corrected *p*-value)	Adjusted or (95% CI)
rs1801133 (C>T)	Dominant						
	CC	45	53	0.024 (0.552)	Ref	0.033 (0.759)	Ref
	CT + TT	31	71		0.51 (0.29–0.92)		0.52 (0.29–0.95)
rs1801133 (C>T)	Genotype						
	CC	45	53	0.014 (0.322)	Ref	0.016 (0.368)	Ref
	CT	22	57	0.556 (1)	0.45 (0.24–0.86)	0.719 (1)	0.45 (0.23–0.86)
	TT	9	14		0.76 (0.30–1.91)		0.84 (0.32–2.18)

C. Failure to MTX monotherapy (12-24 months)							
SNPS	Genotypes	Failure to MTX monotherapy #N = 83	Sustained MTX monotherapy #N = 106	*p*-value (Bonferroni corrected *p*-value)	Or (95% CI)	Adjusted *p*-value (Bonferroni corrected *p*-value)	Adjusted or (95% CI)
rs246240 (A>G)	Recessive						
	AG + AA	79	106	0.036* (0.828)	Ref	-	-
	GG	4	0		12.06 (0.64–227.18)		-
rs2259571 (T>G)	Genotype						
	TT	34	29	0.021 (0.483)	Ref	0.014 (0.322)	Ref
	TG	34	62	0.720 (1)	0.47 (0.25–0.90)	0.786 (1)	0.43 (0.22–0.84)
	GG	15	15		0.85 (0.36–2.04)		0.88 (0.37–2.14)
rs2259571 (T>G)	Dominant						
	TT	34	29	0.049 (1)	Ref	0.040 (0.92)	Ref
	TG + GG	49	77		0.54 (0.30–1.00)		0.52 (0.28–0.97)
rs2274808 (C>T)	Genotype						
	CC	49	77	0.022 (0.506)	Ref	0.024 (0.552)	Ref
	CT	31	23	1.000* (1)	2.12 (1.11–4.05)	0.613 (1)	2.15 (1.11–4.20)
	TT	3	6		0.79 (0.19–3.29)		0.68 (0.16–2.98)
rs1476413 (C>T)	Genotype						
	CC	36	57	0.324 (1)	Ref	0.358 (1)	Ref
	CT	40	47	0.034* (0.782)	1.35 (0.74–2.44)	0.048 (1)	1.32 (0.73–2.41)
	TT	7	2		5.54 (1.09–28.17)		5.24 (1.02–26.95)

A. ACR/EULAR non-remission (6 Months), B. EULAR non-response (4 Months), C. Failure to MTX monotherapy (12–24 months). CI, confidence interval; OR, odds ratio; SNPs, Single nucleotide polymorphisms; Ref, Reference. N, number of patients; MTX, methotrexate; ACR, american college of rheumatology; EULAR, European League Against Rheumatism. Adjusted *p*-value was the *p*-value for each SNP adjusted by smoking history, BMI, gender and age in multivariate logistic regression. *Fisher’s exact test. MTX treatment inefficiency was measured by three criteria and denoted as ACR/EULAR non-remission at 6 months, EULAR non-response at 4 months and Failure to MTX monotherapy from 12 to 24 months. Recessive, dominant and genotype models were applied respectively. The Bonferroni corrected *p*-value was estimated based on hypotheses tested on 23 SNPs to adjust significant results for multiple testing. The corrected *p*-value was shown as ‘1’ if the calculate value >1. The raw *p*-values (without Bonferroni correction adjustment) were also reported because of the exploratory nature of the analyses and to better highlight nuances in the results that would be less visible after correction.

### 3.6 Identification of single nucleotide polymorphisms associated with continuous clinical outcomes post methotrexate monotherapy

**FIGURE 4:**
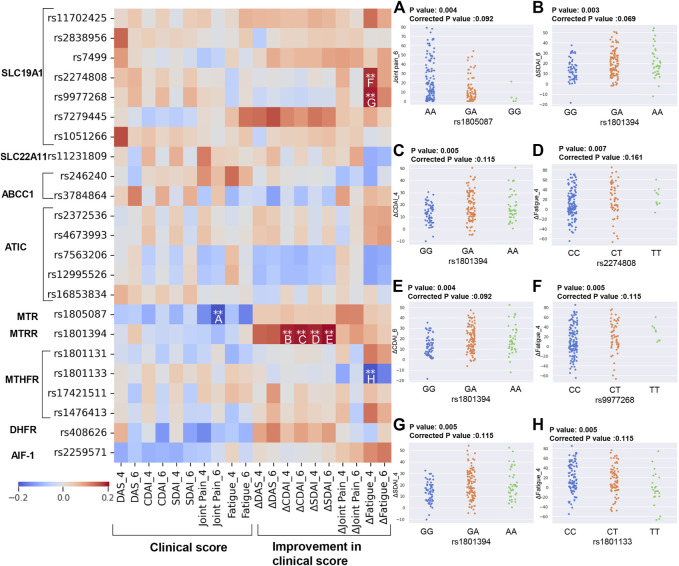
Identification of SNPs correlated to different continuous clinical outcomes. Heatmap showing the correlation between SNPs involved in MTX metabolism and continuous clinical outcomes by Spearman’s rank correlation test. The correlation with significant *p*-value (calculated by Spearman rank **raw *p*-value ≤ 0.01) and relatively high coefficient (absolute value of correlation coefficient >0.18) were marked by asterisk. These significant associations were further shown by scatter plots **(A–H)**, including rs1805087 and joint pain_6 **(A)**, rs1801394 and ΔCDAI_4 **(B)**, rs1801394 and ΔCDAI_6 **(C)**, rs1801394 and ΔSDAI_4 **(D)**, rs1801394 and ΔSDAI_6 **(E)**, rs2274808 and ΔFatigue_4 **(F)**, rs9977268 and ΔFatigue_4 **(G)**, rs1801133 and ΔFatigue_4 **(H)**. The Bonferroni corrected *p*-value was estimated based on hypotheses tested on 23 SNPs to adjust significant results for multiple testing. The raw *p*-values (without Bonferroni correction adjustment) were also reported because of the exploratory nature of the analyses and to better highlight nuances in the results that would be less visible after correction. Delta values means improvements in clinical outcomes from baseline value (baseline value–current value). DAS, Disease activity score; CDAI, Clinical Disease Activity Index; SDAI, Simplified Disease Activity Index.

Disease activity is monitored by DAS, SDAI, CDAI, and patients report outcomes (PRO) capturing features such as joint pain, fatigue. Each SNP was denoted by numbers based on count of minor allele and Spearman’s rank correlation coefficient was estimated for each SNP with these continuous clinical outcomes. Generally, the coefficient for each SNP is low ranging from −0.2 to 0.2 and we further chose the associated one with coefficient >0.18 or < −0.18 for displaying. The heatmap in Figure 4 depicts a negative association between the increasing count of the minor allele G in rs1805087 in *MTR* gene and joint pain at 6 months, which is in line with the trend shown in scatter plot in Figure 4A. By contrast, positive associations were found between the increasing count of the minor allele A of SNP rs1801394 in the *MTRR* gene and four outcomes including improved CDAI and SDAI both at 4 and 6 months. This was in consistent with trends as shown in Figures 4B–E. Additionally, it should be note that the three SNPs, rs2274808 and rs9977268 in the *SLC19A1*, and rs1801133 in the *MTHFR* gene, were all significantly associated with improved fatigue at 4 months (Figure 4 heatmap and F-H). Whereas increasing number of T allele in rs2274808 and rs9977268 were positively correlated with improved fatigue at 4 months, higher number of T allele in rs1801133 were negatively correlated (Figure 4 heatmap and F-H). As the same case in the analyses for categorical outcomes, the correlations between SNPs and outcomes became insignificant with Bonferroni correction.

**FIGURE 5:**
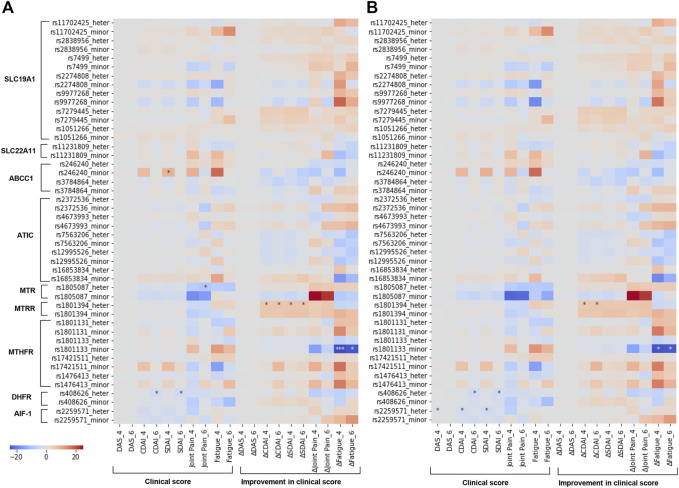
Identifying associated SNPs with continuous clinical outcomes by linear regression. Heatmap showing the association between SNPs involved in MTX metabolism and continuous clinical outcomes by linear regression with ordinary least squares regression (OLS). Coefficient and *p*-value shown in heatmap **(A,B)** were respectively estimated by univariate and multivariate regression analysis with age, BMI, gender and smoking as covariates. Significance level was corrected by Bonferroni correction for multiple testing and significant associations were marked by asterisk (*p-value ≤ 0.05, ***p-value ≤ 0.001). Delta values means improvements in clinical outcomes from baseline value (baseline value–current value). DAS, Disease activity score; CDAI, Clinical Disease Activity Index; SDAI, Simplified Disease Activity Index.

Next, we further investigated the association between SNP and clinical continuous outcomes by using linear regression with ordinary least squares (OLS) in both univariate model and multivariate model (Figures 5A,B). Based on Bonferroni corrected significance level, both models identified significant positive association between rs1801394_heter (GA) and improved CDAI at 4 and 6 months, which was in line with the result in Spearman’s correlation analysis. Moreover, the negative association between rs1801133_minor (TT) and improved fatigue at 4 and 6 months, as well as the negative association between rs408626_heter (AG) and CDAI/SDAI at 6 months were identified by both univariate and multivariate analysis. Of note, after adjustment by covariates, rs2259571_heter (TG) become significantly associated with disease activities, including DAS, CDAI and SDAI at 4 months. (Figure 5B).

## 4 Discussion

Despite the long-term use of MTX for patients with RA, our ability to predict clinical therapeutic response to MTX is limited. Recently, studies of association between MTX metabolism related- SNPs and therapeutic response demonstrated their involvement in interpatient variability in clinical response. Here a comprehensive analysis of SNPs in genes encoding proteins for MTX uptake and metabolism and their association with multiple clinical outcomes post MTX treatment was studied in patients with RA. The disclosed SNPs include rs1801394, rs408626 and rs2259571 associated with disease activity relevant continuous outcomes, as well as rs1801133 associated with improved fatigue. Additionally, potential associations between two SNPs, rs1476413, rs3784864 and ACR/EULAR non-remission; between one SNP rs1801133 and EULAR response; between four SNPs rs246240, rs2259571, rs2274808, rs1476413 and failure to MTX monotherapy after 12–24 months were identified, despite insignificant when Bonferroni correction applied.

The increased number of minor allele A in rs1801394 G>A in the *MTRR* was demonstrated to be associated with more improved CDAI at 4 and 6 months by Spearman’s rank correlation test. This was further supported by both univariate and multivariate linear regression analysis showing rs1801394_GA significantly associated with more improved CDAI with Bonferroni corrected *p*-value as compared with GG. The similar trend can be also observed in rs1801394_AA from Figures 4B,C, despite no statistical significance found due to limited number of participants. This concluded a favorable role of the allele A in this SNP associated with positive continuous outcomes after MTX treatment. Our results were in contrast to one study on 915 European Caucasian origin patients with RA where A allele was associated with less improvement than the G allele according to ΔDAS28 (*p* = 0.0016) and EULAR response (*p* = 0.004) (López-Rodríguez et al., 2018). Additionally, other studies on Chinese and Indian population identified no association between the SNP and MTX response (Ghodke-Puranik et al., 2015; Lv et al., 2018). Due to difference in the analyzed population, a clear conclusion would be precluded. More studies on Nordic population remain needed for validation.

Another disease activity associated SNP is rs2259571 TG in *AIF-1* gene which was found to be significantly associated with low level of DAS, CDAI and SDAI at 4 months. Moreover, The T allele and TG genotype were both shown to be negatively associated with failure to MTX monotherapy, despite at an uncorrected significance level. AIF-1 is a cytoplasmic, inflammation-responsive protein and Pawlik and coworkers demonstrated that the patients with the *AIF-1* rs2259571 GG genotype have a poorer response (DAS28 > 2.4 at 6 months) to MTX treatment by studying 221 Poland patients with RA (*p* = 0.03, OR = 2.44) (Pawlik et al., 2013). This was in contrast to the present results which demonstrated that G allele was associated with more sustained MTX monotherapy and low level of disease activity. Our result was also supported by machine learning analysis in the other paper by us identifying rs2259571 TG as predictor for more sustained MTX monotherapy. The inconsistency in conclusion from other study may result from the different definition of response outcome and distinct population for analysis. Our result stressed important role of rs2259571 in predicting MTX related outcome and more research is warranted for further investigation of this SNP. In addition to rs1801394 and rs2259571, rs408626 AG genotype in *DHFR* gene was identified to be associated with low value of CDAI and SDAI at 6 months, this is in contrast to other study where AA genotype was associated with the less reduction in relative DAS28 score (*p* = 0.05) (Milic et al., 2012).

The TT genotype of rs1801133 in the *MTHFR* gene which was negatively associated with improvement in fatigue at 4 months post MTX monotherapy. As this was shown both through Spearman’s rank correlation test and the univariate and multivariate linear regression with Bonferroni corrected *p*-value, suggesting for a potent role in predicting fatigue by screening for this allele variant. Moreover, this SNP was further associated with EULAR response outcome in the dominant and genotype-heterozygote model at an uncorrected significance level. This may implicate that population carrying T allele (CT and TT) or CT may have higher proportion of responders as compared with those carrying the CC genotype. Recent studies on this SNP showed inconsistent result. Whereas a study from Poland (*n* = 174) demonstrated a similar result as the present study that T allele was shown to be associated with improved remission outcomes (*p* = 0.02, OR = 0.46) (Kurzawski et al., 2007), a different study on 233 Portuguese patients found that TT were associated with increased risk for non-response to MTX defined by DAS28< =3.2 (*p* = 0.013, OR = 4.63) (Lima et al., 2014). Moreover, studies on Asian population including 217 Indian RA population, 110 Chinese and 67 Pakistani showed no association between the SNP and treatment outcomes (Xiao et al., 2010; Ghodke-Puranik et al., 2015; Iqbal et al., 2015). Due to conflicted results from different studies on different ethnical population, a clear conclusion was precluded and more studies on Norwegian population are needed.

The *MTHFR* gene was further associated with both non-remission post MTX treatment and failure to sustained MTX monotherapy through TT genotype of SNP rs1476413 C>T, at an uncorrected significance level. The *MTHFR* gene encodes an enzyme responsible for thymidine and methionine synthesis, which are one of targets of MTX (Figure 1) (Owen et al., 2013b). The association of rs1476413 TT with failure in responding to MTX treatment is consistent with a study by Salazar and coworkers who identified that T allele was associated with a poor response to MTX treatment in 124 Spanish patients with RA (*p* = 0.0086, OR = 3.56) (Salazar et al., 2014), despite difference in the outcome definition.

The SNP rs3784864 AA in *ABCC1* gene in the ABC encoding gene was associated with ACR/EULAR non-remission at 6 months at an uncorrected significance. The ABC gene family are MTX transporters responsible for MTX efflux. The gene *ABCC1* is located on chromosome 16p13 and the SNP rs3784864 is characterized by an intronic G to an A substitution (Haga and Burke, 2004). Our study found that population with AA was associated with non-remission. This observation is in contrast to a recent report demonstrating that MTX non-response was associated with G carriers of rs3784864 in 233 Portugal patients with RA (*p* = 0.015, OR = 4.24) (Lima et al., 2015). Different from Boolean ACR/EULAR Remission as the outcome used in this study, they defined non-response as DAS28 > 3.2 in two consecutive evaluations with 3 months as each interval evaluation. These different ethical backgrounds of population and outcome definitions in these studies may be the reason for this inconsistency in results.

Moreover, the SNP rs246240 GG in *ABCC1* and rs2274808 CT in *SLC19A1* was positively associated with failure to sustain MTX monotherapy at an uncorrected significance level, suggesting that populations with genotype rs246240 GG and rs2274808 CT might have a higher risk of MTX withdrawal before 12 months. Such a role for rs246240 has been validated by Lima and coworkers in a multivariate genotype analysis which showed that G carriers are associated with poor response to MTX in 233 Portugal population (*p* = 0.008, OR = 5.47) (Lima et al., 2015). By contrast, the role for rs2274808 is inconsistent with other study: A study in 248 United Kingdom population found the T allele associated with failure to MTX treatment (*p* = 0.009, OR = 1.76) (Owen et al., 2013a), whereas in the present study rs2274808 CT and not TT was found to be associated with early discontinuation of MTX monotherapy compared to RA patients carrying the CC genotype.

The main strengths our study include 1) high quality of data: all patients were naïve to DMARDs and initiated with MTX as first-line therapy, with the studied population being highly homogenous regarding ethnic origin and thus representative; 2) a comprehensive assessment of 23 SNPs associated with MTX metabolism and inflammatory response as well as a variety of relevant outcome measures 3) a rigorous statistical evaluation taking into consideration common potential cofounders including age, BMI, smoking and gender, with Bonferroni correction for multiple testing to avoid false positive findings. Nevertheless, several potential limitations should be noted. First, the sample size of our present study was relatively small (*n* = 200), thus statistical power was relatively limited to detect variants with weak effect sizes. Secondly, there is a possibility of false negative rate caused by Bonferroni correction, which may filter some truly significantly associated SNPs. To this end, we also reported uncorrected raw *p*-value for analyses on association for categorical outcomes and on Spearman rank correlation for continuous outcomes.

## 5 Conclusion

In summary, we have identified and validated several SNPs associated with a variety of clinical outcomes post MTX monotherapy in a Norwegian population with early DMARD naïve RA. Among these SNPs, rs1801394, rs408626, and rs2259571 are significantly associated with disease activity relevant continuous outcomes, as well as rs1801133 associated with improved fatigue. Moreover, rs1476413 and rs246240 had been found associated with categorical outcomes of ACR/EULAR non-remission and failure to MTX monotherapy respectively, which was identical to previous studies and thus strengthened reliability of these findings. Despite this, more investigation should be conducted to validate the results in other cohorts before these may be applied in clinical decision-making.

## Data Availability

The original contributions presented in the study are included in the article/Supplementary Material, further inquiries can be directed to the corresponding author.
