# Supplementary material for: Identification of SNPs associated with methotrexate treatment outcomes in patients with early rheumatoid arthritis
Source: Front Pharmacol. 2022 Nov 17;13:1075603. doi: 10.3389/fphar.2022.1075603 (PMC9714492; doi:10.3389/fphar.2022.1075603)
Supplement: Supplementary file 1 [file DataSheet1.docx]

Supplementary Material

## Supplementary Table 1: DAS-based EULAR response

|  | ***Change from baseline DAS*** | | |
| --- | --- | --- | --- |
| **DAS at time-point** | ΔDAS ≤ -1.2 | -1.2 < DAS < -1.2 | ΔDAS ≥ 0.6 |
| DAS **≤ 2.4** | Good | Moderate | None |
| **2.4 < DAS ≤ 3.7** | Moderate | Moderate | None |
| **DAS > 3.7** | Moderate | None | None |

The European League Against Rheumatism (EULAR) response rates was calculated. The DAS-based EULAR response is defined by the state and change in DAS, and categorized into good, moderate and none using the above definitions.

| **Gene** | **SNPs** | **Study results** | **P value, OR (95% CI)** | **Reference**  **(PMID)** |
| --- | --- | --- | --- | --- |
| ***SLC19A1*** | 11702425 [T>C] | TT allele: No response | 0.001, 1.86 (1.26-2.74) | 22450926 |
|  | 2838956 [A>G] | G allele: No response  allele: A vs G;  Recessive: GG vs AG+AA | 0.02, 1.366 (1.051-1.776)  0.03, 0.592 (0.361-0.969) | 28266606 |
|  | 7499 [G>A] | A allele: No response | 0.02, 1.50 (1.00-2.19) | 22450926 |
|  | 2274808 [C>T] | T allele: No response | 0.009, 1.76 (1.17-2.67) | 22450926 |
|  | 9977268 [C>T] | T allele: No response | 0.02, 1.67 (1.08-2.58) | 22450926 |
|  | 7279445 [C>T] | T allele: No response | 0.05, 1.44 (0.99-2.08) | 22450926 |
|  | 1051266 [G>A] | AA: Response compared to AG and GG | 0.02, 3.32 (1.26-8.79) | 17325736 |
| ***SLC22A11*** | 11231809 [T>A] | T carrier: No response | 0.03, 5.37 (1.17–24.71) | 26086825 |
| ***ABCC1*** | 246240 [A>G] | G carrier: No response | 0.008, 5.47 (1.56–19.25) | 26086825 |
|  | 3784864 [G>A] | G carrier: No response | 0.01, 0.24 (0.07-0.76) | 26086825 |
| ***ATIC*** | 2372536 [C>G] | CC/CG carrier: Poor response | 0.03, 3.61 (1.15-11.34) | 19902562 |
|  | 4673993 [T>C] | T carrier: No response | 0.013, 5.16 (1.42–18.76) | 24967362 |
|  | 7563206 [C>T] | T allele: No response | 0.01,1.60 (1.10–2.33) | 22450926 |
|  | 12995526 [C>T] | C allele: No response | 0.01, 1.65 (1.13–2.42) | 22450926 |
|  | 16853834 [C>T] | T allele: No response | 0.04, 1.70 (1.02–2.82) | 22450926 |
| ***MTR*** | 1805087 [A>G] | AG: No response | 0.03, 0.19 (0.04-0.88) | 28266606 |
| ***MTRR*** | 1801394 [G>A] | AG/GG: weaker efficacy | 0.03, 0.19 (0.04-0.88) | 28266606 |
| ***MTHFR*** | 1801131 [A>C] | CC-AC: Response | 0.01, 3.53 (1.18–10.58) | 20863444 |
|  | 1801133 [C>T] | TT: No response | 0.015, 3.08 (1.21–7.84) | 24967362 |
|  | 17421511 [G>A] | GG: Response | 0.04, 5.34 (0.42–68.40) | 25084201 |
|  | 1476413 [C>T] | CC carrier: Response | 0.024, 3.35 (1.10–10.24) | 25084201 |
| ***DHFR*** | 408626 [A>G] | AA carrier: No response | 0.05 (not recorded) | 22324981 |
| ***AIF-1*** | rs2259571 [T>G] | GG: No response | 0.03, 0.41 (0.18-0.92) | 24018427 |

**Supplementary Table 2: Studies reporting associations of polymorphisms with response to MTX in RA.**

Solute Carrier Family 19 Member 1 (*SLC19A1*); Solute Carrier Family 22 Member 11 (*SLC22A11*) and ATP Binding Cassette Subfamily C Member 1(*ABCC1); ATIC* (5-aminoimidazole-4-carboxamide ribonucleotide transformylase (*ATIC*) gene; 5-Methyltetrahydrofolate-Homocysteine Methyltransferase (*MTR*); Methionine Synthase Reductase (*MTRR*) gene; methylenetetrahydrofolate reductase (*MTHFR*)**;** Dihydrofolate reductase (*DHFR*) gene and Allograft inflammatory factor 1 (*AIF*-1).

**
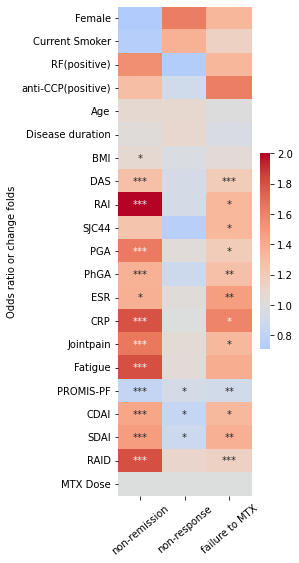
**

**Supplementary Figure 1:** Heatmap showing odds ratio (for categorical characteristics) and value changes (for continuous characteristics) of each characteristic between subgroups. The two subgroups were defined according to ACR/EULAR remission criteria at 6 months (non-remission), response criteria at 4 months (non-response) and failure to MTX monotherapy from 12 to 24 months (failure to MTX). P value was calculated by chi-square test and Mann-Whitney U test for categorical and continuous characteristics respectively (*P value ≤0.05, **P value ≤0.01, ***P value ≤0.001). Abbreviations: RF=Rheumatoid factor; Anti-CCP= Anti-cyclic citrullinated protein; Disease duration (months) = Disease duration in months; BMI=Body Mass Index; DAS=Disease Activity scores; RAI= Ritchie Articular Index; SJC44=Swollen joint count based on 44 joints; PGA= Patients global assessment score; PhGA=Physician global assessment score; ESR=Erythrocyte Sedimentation rate; CRP=C reactive protein; PROMIS-PF: Patients reported outcomes measurement information physical function T-score; CDAI=Clinical disease activity index; SDAI=Simplified Disease Activity Index; RAID=RA impact disease score; MTX dose= Dose of methotrexate/week.

**Supplementary Table 3: Associations of polymorphisms with ACR/EULAR non-remission status post treatment.**

| **ACR/EULAR non-remission** | P value  recessive model | P value  dominant model | P value genotype model (heter) | P value genotype model (minor) |
| --- | --- | --- | --- | --- |
| **rs11231809** | 0,216 | 0,676 | 0,957 | 0,252 |
| **rs11702425** | 0,763 | 0,542 | 0,454 | 0,922 |
| **rs12995526** | 0,571 | 0,855 | 0,998 | 0,634 |
| **rs16853834** | 0,389 | 0,602 | 0,794 | 0,376 |
| **rs17421511** | 0,163 | 0,696 | 0,967 | 0,163 |
| **rs1801131** | 0,287 | 0,74 | 0,549 | 0,376 |
| **rs1801133** | 0,366 | 0,503 | 0,706 | 0,33 |
| **rs1801394** | 0,051 | 0,829 | 0,619 | 0,145 |
| **rs1805087** | 0,327 | 0,511 | 0,622 | 0,304 |
| **rs2259571** | 0,364 | 0,971 | 0,791 | 0,477 |
| **rs2274808** | 0,098 | 0,634 | 0,312 | 0,135 |
| **rs2372536** | 0,684 | 0,722 | 0,818 | 0,65 |
| **rs246240** | 0,75 | 0,429 | 0,46 | 0,708 |
| **rs2838956** | 0,986 | 0,804 | 0,796 | 0,893 |
| **rs4673993** | 0,684 | 0,831 | 0,936 | 0,679 |
| **rs7279445** | 0,512 | 0,805 | 0,975 | 0,577 |
| **rs7499** | 0,918 | 0,643 | 0,596 | 0,911 |
| **rs7563206** | 0,571 | 0,733 | 0,87 | 0,572 |
| **rs9977268** | 0,111 | 0,533 | 0,273 | 0,146 |
| **rs408626** | 0,631 | 0,563 | 0,438 | 0,997 |
| **rs1051266** | 0,671 | 0,804 | 0,679 | 0,846 |

**Supplementary Table 4: Associations of polymorphisms with DAS based EULAR non-response post treatment.**

| **DAS based EULAR non-response** | P value  recessive  model | P value  dominant  model | P value genotype  model (heter) | P value genotype  model (minor) |
| --- | --- | --- | --- | --- |
| **rs11231809** | 0,569 | 0,403 | 0,488 | 0,415 |
| **rs11702425** | 0,518 | 0,431 | 0,545 | 0,435 |
| **rs12995526** | 0,817 | 0,521 | 0,454 | 0,838 |
| **rs1476413** | 0,193 | 0,73 | 0,494 | 0,272 |
| **rs16853834** | 0,12 | 0,747 | 0,857 | 0,127 |
| **rs17421511** | 0,736 | 0,838 | 0,758 | 0,763 |
| **rs1801131** | 0,276 | 0,996 | 0,772 | 0,339 |
| **rs1801394** | 0,276 | 0,249 | 0,416 | 0,18 |
| **rs1805087** | 0,697 | 0,073 | 0,08 | 0,571 |
| **rs2259571** | 0,492 | 0,325 | 0,205 | 0,922 |
| **rs2274808** | 0,247 | 0,236 | 0,107 | 0,349 |
| **rs2372536** | 0,849 | 0,423 | 0,357 | 0,945 |
| **rs246240** | 0,096 | 0,149 | 0,309 | 0,081 |
| **rs2838956** | 0,275 | 0,696 | 0,983 | 0,322 |
| **rs3784864** | 0,061 | 0,491 | 0,964 | 0,112 |
| **rs4673993** | 0,849 | 0,554 | 0,486 | 0,991 |
| **rs7279445** | 0,722 | 0,498 | 0,555 | 0,538 |
| **rs7499** | 0,295 | 0,856 | 0,873 | 0,366 |
| **rs7563206** | 0,817 | 0,685 | 0,615 | 0,945 |
| **rs9977268** | 0,545 | 0,531 | 0,412 | 0,621 |
| **rs408626** | 0,702 | 0,201 | 0,139 | 0,641 |
| **rs1051266** | 0,388 | 0,423 | 0,579 | 0,309 |

**Supplementary Table 5: Associations of polymorphisms with failure to sustained MTX monotherapy.**

| **Failure to sustained MTX monotherapy** | P value  recessive  model | P value  dominant  model | P value genotype  model (heter) | P value genotype  model (minor) |
| --- | --- | --- | --- | --- |
| **rs11231809** | 0,753 | 0,995 | 0,918 | 0,814 |
| **rs11702425** | 0,784 | 0,229 | 0,169 | 0,939 |
| **rs12995526** | 0,955 | 0,298 | 0,28 | 0,528 |
| **rs16853834** | 0,832 | 0,412 | 0,352 | 0,914 |
| **rs17421511** | 0,099 | 0,937 | 0,656 | 0,108 |
| **rs1801131** | 0,174 | 0,077 | 0,13 | 0,089 |
| **rs1801133** | 0,684 | 0,882 | 0,994 | 0,693 |
| **rs1801394** | 0,267 | 0,945 | 0,637 | 0,438 |
| **rs1805087** | 0,365 | 0,297 | 0,368 | 0,318 |
| **rs2372536** | 0,897 | 0,454 | 0,456 | 0,752 |
| **rs2838956** | 0,733 | 0,512 | 0,405 | 0,94 |
| **rs3784864** | 0,373 | 0,931 | 0,683 | 0,581 |
| **rs4673993** | 0,897 | 0,574 | 0,583 | 0,789 |
| **rs7279445** | 0,284 | 0,741 | 0,977 | 0,37 |
| **rs7499** | 0,89 | 0,485 | 0,488 | 0,692 |
| **rs7563206** | 0,955 | 0,408 | 0,393 | 0,614 |
| **rs9977268** | 0,287 | 0,127 | 0,059 | 0,415 |
| **rs408626** | 0,356 | 0,161 | 0,241 | 0,158 |
| **rs1051266** | 0,771 | 0,512 | 0,42 | 0,921 |
